# Gradual Restraint Habituation for Awake Functional Magnetic Resonance Imaging Combined With a Sparse Imaging Paradigm Reduces Motion Artifacts and Stress Levels in Rodents

**DOI:** 10.3389/fnins.2021.805679

**Published:** 2021-12-21

**Authors:** Gabriele Russo, Xavier Helluy, Mehdi Behroozi, Denise Manahan-Vaughan

**Affiliations:** ^1^Department of Neurophysiology, Medical Faculty, Ruhr University Bochum, Bochum, Germany; ^2^International Graduate School of Neuroscience, Ruhr University Bochum, Bochum, Germany; ^3^Department of Biopsychology, Institute of Cognitive Neuroscience, Faculty of Psychology, Ruhr University Bochum, Bochum, Germany

**Keywords:** learning, rodent, auditory, attention, stress, corticosterone, awake fMRI, functional magnetic resonance imaging

## Abstract

Functional magnetic resonance imaging, as a non-invasive technique, offers unique opportunities to assess brain function and connectivity under a broad range of applications, ranging from passive sensory stimulation to high-level cognitive abilities, in awake animals. This approach is confounded, however, by the fact that physical restraint and loud unpredictable acoustic noise must inevitably accompany fMRI recordings. These factors induce marked stress in rodents, and stress-related elevations of corticosterone levels are known to alter information processing and cognition in the rodent. Here, we propose a habituation strategy that spans specific stages of adaptation to restraint, MRI noise, and confinement stress in awake rats and circumvents the need for surgical head restraint. This habituation protocol results in stress levels during awake fMRI that do not differ from pre-handling levels and enables stable image acquisition with very low motion artifacts. For this, rats were gradually trained over a period of three weeks and eighteen training sessions. Stress levels were assessed by analysis of fecal corticosterone metabolite levels and breathing rates. We observed significant drops in stress levels to below pre-handling levels at the end of the habituation procedure. During fMRI in awake rats, after the conclusion of habituation and using a non-invasive head-fixation device, breathing was stable and head motion artifacts were minimal. A task-based fMRI experiment, using acoustic stimulation, conducted 2 days after the end of habituation, resulted in precise whole brain mapping of BOLD signals in the brain, with clear delineation of the expected auditory-related structures. The active discrimination by the animals of the acoustic stimuli from the backdrop of scanner noise was corroborated by significant increases in BOLD signals in the thalamus and reticular formation. Taken together, these data show that effective habituation to awake fMRI can be achieved by gradual and incremental acclimatization to the experimental conditions. Subsequent BOLD recordings, even during superimposed acoustic stimulation, reflect low stress-levels, low motion and a corresponding high-quality image acquisition. Furthermore, BOLD signals obtained during fMRI indicate that effective habituation facilitates selective attention to sensory stimuli that can in turn support the discrimination of cognitive processes in the absence of stress confounds.

## Introduction

Functional magnetic resonance imaging (fMRI), based on the blood-oxygenation-level dependent (BOLD) signal, is a powerful non-invasive procedure, which serves as an indirect indicator of neuronal activity (Ogawa et al., 1992; Logothetis, 2002). This method has been already used to examine brain connectivity and to map specific neuronal functions in humans and animals (Ogawa et al., 1992; Canals et al., 2009; Angenstein, 2019; Behroozi et al., 2020). Animal studies are particularly helpful in relating mechanistic observations to functional patterns with regard to the scrutiny of cognition and information processing (Strauch et al., 2021). To acquire meaningful functional neuroimaging data, subjects must remain still during imaging. For this reason, most animal fMRI studies are conducted under general anesthesia, or sedation, to minimize motion artifacts and to remove the stress component (Sicard et al., 2003): Stress not only increases the likelihood of head movement but also distorts cognition and information encoding (Lucassen et al., 2014; Joëls, 2018), selective attention (Elling et al., 2011; Dagnino-Subiabre, 2013; Hurtubise and Howland, 2017), and creates a bias in the fMRI results obtained (Diamond et al., 1992, 2004; Kim and Diamond, 2002). Unfortunately, the use of anesthesia, as a putative alternative strategy for fMRI in rodents, not only profoundly alters brain functions related to emotion and cognition during MRI, but can also influence global cerebrovascular reactivity and potently affect the magnitude of the BOLD signal through different effects on cerebral blood flow (CBF), cerebral blood volume (CBV), and the oxygenation ratio (Lahti et al., 1999; Sicard et al., 2003; Reed et al., 2013). Anesthesia and sedation also alter the threshold for the induction of synaptic plasticity (Riedel et al., 1994; Ribeiro et al., 2015), a key cellular process underlying experience-dependent information encoding and updating in the brain (Manahan-Vaughan, 2017).

Recently, to overcome the confounds generated by the use of anesthesia, awake fMRI protocols have been developed to enable imaging in animals such as non-human primates, rodent, and birds (Lahti et al., 1998; King et al., 2005; Goense and Logothetis, 2008; Desai et al., 2011; Behroozi et al., 2020). These procedures inevitably require that the animals undergo body and head restraint, as well as exposure to the unpredictable and loud acoustic noise of the scanner that can reach sound levels of up to 81 dB (Cheung et al., 2012). Although physical restraint is currently an irreplaceable tool to minimize motion, and acoustic noise intrinsically accompanies fMRI recordings, both are potent stressors for rodents (Porro and Carli, 1988; Khasar et al., 2005; Low et al., 2016). Furthermore, both are used to create animal models of chronic stress (Kugler et al., 1990; Van raaij et al., 1996; Khasar et al., 2005; Anisman et al., 2007) and even of clinical depression (Pittenger and Duman, 2008; Li et al., 2018; Huang et al., 2019; Seewoo et al., 2020). Cognition-related brain activation seen in fMRI data is very likely to be confounded by factors such as restraint-induced and noise-related stress (Pavlides et al., 2002; Cerqueira et al., 2007; Maggio and Segal, 2007; Patel et al., 2012). Accordingly, it was shown that chronic stress impacts large-scale functional connectivity networks in the rat brain, resulting in an increase in connectivity in somatosensory, visual, and default mode networks (Henckens et al., 2015).

Current protocols comprise habituation of the animals *directly* to the final experimental conditions, with the goal of reducing the stress associated with head and body fixation. Typically, these approaches do not incorporate a gradual acclimatization to the scanning procedures; rather they usually involve short-term habituation to head-fixation in a scanner, or scanner-like environment. In addition to it serving as a stressor, the loud intermittent noise of varying intensities that is a characteristic of fMRI acquisition can also limit the scrutiny of certain brain processes such as audition and cognition (Novitski et al., 2003; Scarff et al., 2004; Haller et al., 2005; Tomasi et al., 2005), as well as affect selective attention (Elling et al., 2011). Thus, a strategy that permits effective habituation to awake fMRI, not only will serve to minimize stress levels and associated motion artifacts, but will also facilitate the accurate interpretation of BOLD images obtained during sensory and cognitive information processing.

In the present study, we describe a novel method to habituate rats to fMRI procedures that results in lower stress levels in the animals, such that stress confounds in data acquisition are circumvented. In addition, a sparse MRI sequence was designed to limit the scanner noise exposure of the animals and that enabled acoustic stimulation to elicit clear BOLD signals in auditory structures of the brain. This double approach (improved habituation procedure and sparse fMRI acquisition, tuned to lower acoustic stress) provides a reliable basis through which stress-related biases in data acquisition can be avoided during fMRI in awake rats. Furthermore, we report that patterned acoustic stimulation at 70 dB results in activation of multiple auditory structures that are known to engage in sound perception and interpretation. Here, animals exhibited an ability to discriminate the acoustic stimulation from the backdrop of scanner noise, demonstrating focused attention that corresponded to BOLD signal elevations in the thalamus and reticular formation.

## Materials and Methods

### Animals

At the beginning of the study, four-week-old male Hooded Lister rats (Charles River Breeding Laboratories, Germany, *n* = 6) were group-housed in type IV cages (600 × 380 × 195 mm), in standard conditions (temperature, 22 ± 2°C; humidity 55 ± 5%; 12:12-h day/night cycle, light on at 6 a.m.) in specialized animal housing units (Zoonlab, Castrop-Rauxel, Germany). Water was available *ad libitum*. The food amount was limited in the home cages (∼18g of pellets per day for each rat). This was because additional food was provided by the experimenter, by hand to the animals, during the habituation trials. Weight as monitored on a daily basis (Supplementary Table 1).

All procedures were performed according to the guidelines of the European Communities Council Directive of September 22nd, 2010 (2010/63/EU) for care of laboratory animals and after approval of the ethics committee of the federal state of NorthRhine Westphalia (NRW) (Landesamt für Naturschutz, Umweltschultz und Verbraucherschutz, NRW, Bezirksamt Arnsberg). All efforts were made to minimize the number of rats used for this study.

### Habituation Strategy

#### Apparatus

The goal of habituation was to acclimatize the animals to head-restraint during awake fMRI. The head restraint device we used did not involve surgical implantation of an anchor socket, as used by others (Miller et al., 2003; Sachdev et al., 2003; Tsurugizawa et al., 2010, 2012; Hung et al., 2015; Chang et al., 2016; Roh et al., 2016; Behroozi et al., 2018). Rather, we constructed a device based on the non-invasive head restraint system described by Stenroos and colleagues (Stenroos et al., 2018). We used a 3D printer (Formlabs, Form3, Berlin, Germany) (printing resolution: 25 μm) to print the parts using RS-F2-GPCL-04 clear resin (Formlabs, Berlin, Germany).

During the handling and body restraint phases, the illumination of the room was exclusively produced by electrical lighting (500 Lux, no natural light from outside). This was done in order to have constant light conditions. For the other phases, where a mock scanner environment was recreated (i.e., from the habituation steps involving acclimatization to the darkness until the head fixation habituation) the only source of light in the room was a 11 W red-colored light bulb (Osram, Berlin, Germany) positioned on the experimental table approximately 60 cm away from the animal. The MRI environment was mimicked using a rat bed (Pharmascan rat bed base T12554 and rat brain/body tip SUC T12560, Bruker Biospin, Ettlingen, Germany), at the bottom of which a thick layer of foam plastic was taped. This was done in order to absorb possible vibrations generated by the MRI device and body motion, since during the actual fMRI recordings, the animal bed was placed on the resonator to circumvent that the animal’s motion created a large oscillatory effect. To mimic the dark MRI environment, a plastic tube (internal diameter: 80 mm), covered in transparent-red film was used. The acoustical noise of the imaging sequences was recorded using a microphone (ULTRAMIC250K, Dodotronic, Italy) positioned ca. 1 m in front of the magnet bore. During habituation, this noise was played via a loudspeaker (Kilburn speaker, Marshall Amplification PLC, Milton Keynes, United Kingdom) positioned 50 cm in front of the plastic tube bore. The volume, bass and treble levels of the speaker were adjusted to produce a sound output as similar as possible to the MRI noise (70 dB in intensity).

#### Habituation Procedure

**FIGURE 1:**
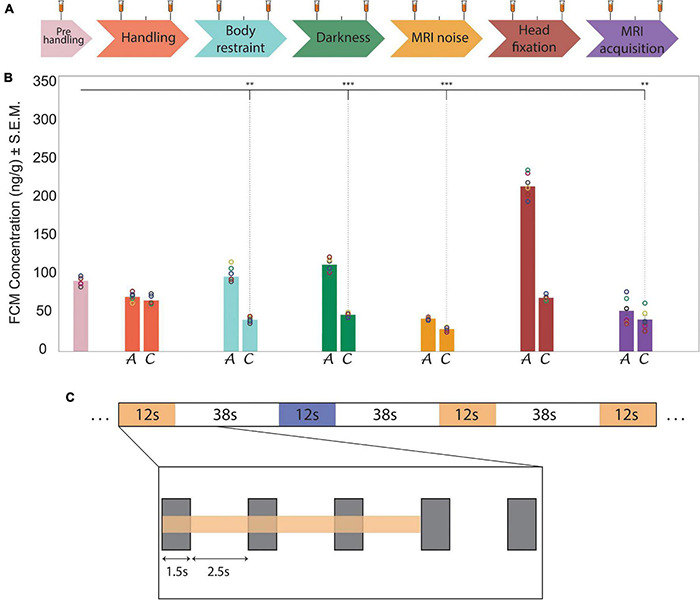
Habituation protocol, stress level indicators of fecal corticosterone metabolites and experimental design of the auditory fMRI task.**(A)** Timeline representation of the habituation protocol that was designed to enable stable recordings during awake fMRI. (tick, 24 h; white space, 48 h break; long tick with test tube, stress sample collection, short tick, habituation without stress sample collection). **(B)** Bar charts show results from fecal corticosterone metabolite (FCM) assessments (*n* = 6). Circles indicate the single values obtained in the animals for each evaluation step. The bar chart colors correspond to the habituation and recording steps shown in the timeline in part **(A)**. In each step, bar chart A reports the acute FCM levels and bar chart C reports the chronic FCM levels. FCM concentration is expressed as ng of FCM per g of fecal matter. Significance (One Way ANOVA) vs. pre-handling levels: ^**^p ≤ 0.01, ^***^p ≤ 0.001. During the last phase of the habituation protocol FCM levels are significantly lower than pre-handling levels (*t*(5) ≥ 5.25, *p* < 0.03, *n* = 6). **(C)** Schematic representation of the auditory task. The two acoustic stimulation patterns (signified in yellow and blue) were randomly distributed during the sequence acquisition. A closer look at one sound stimulation (inlay) shows the duration of the scanner noise (gray rectangles) - relative to the slice acquisition period (TA = 1.5 s). This occurred at the beginning of each TR and was followed by a quiet period (no slice acquisition noise) of 2.5 s.

Each phase of the habituation involved three sessions, experienced at the rate of one per day for three days, here referred as days 1, 2 and 3. Day 1 of each habituation phase reflects the state of acute experience and day 3 reflects the state of chronic experience. The habituation session durations for each kind of manipulation, lasted 20 min on day 1, 40 min on day 2 and 60 min on day 3. Handling was always conducted for 20 min. After the completion of each habituation phase, the rats experienced a resting period of 48 h during which no manipulations occurred. The total duration of the habituation procedure was 3 weeks (Figure 1A), and all the procedures were always carried out at the same time of the day (6 a.m.–12 p.m.) to minimize the impact of circadian rhythm variations (Stephan, 1983; Weibel et al., 2002).

The first habituation phase comprised “handling,” during which the animals were habituated to the experimenter and learned to associate their presence with a positive experience (Figure 1A). For this reason, during the handling procedures, all possible efforts were made to alternate between basic handling and the mimicry of aspects of the animals’ playful rough-and-tumble behavior (Cloutier et al., 2018). Animals were also given chocolate chips as treats (chocolate sprinkles, De Ruijter, Amsterdam, Netherlands).

The second phase consisted of physical restraint of the animal (Figure 1A). During this initial body restraint, the paws were gently taped with masking tape (30 mm, Tesa, Norderstedt, Germany). The front paws were positioned alongside the body in the direction of the tail and another piece of masking tape further secured the paws, bridging them on top of the body. The hind paws were first gently taped together and then, with a second piece of masking tape, they were taped to the tail. A sheet of foam plastic (thickness = 2 mm) was used to wrap the animal from the shoulders to the hind paws. After this the rat was positioned in the rat bed inside the film-covered plastic tube. The procedure was repeated for 3 consecutive days, increasing the habituation time from 20 to 60 min. After the third day the animals were not manipulated for the next 48 h.

The third habituation phase added to the body restraint the darkness condition (Figure 1A). The rats were habituated, as described for the previous phase, with the difference being that now the only light in the room was a red light (to allow the experimenter to check on the animal). The wavelength of the red light bulb used (∼700 nm) is outside the range of effective visible light for rodents (Wang et al., 2011; Nikbakht and Diamond, 2021), thus for the rats, the habituation was conducted ostensibly in darkness.

During the fourth habituation step MRI noise was added to the previous conditions (Figure 1A).

During the last phase of the habituation procedure in the mock scanner environment, the animal experienced head fixation for the first time (Figure 1A). Whereas, in the original protocol by Stenroos et al. (2018), anesthesia was applied on each habituation day, our adapted procedures included the use of anesthesia *only* from this stage onward. During the head-fixation, the volatile anesthetic (Isoflurane CP 1 ml/ml, CP-Pharma, Germany) was applied in a carrier gas with a flow of 1L_*air*_/min (1.5% anesthesia via a snout mask) for at least 5 min. The anesthesia was gradually reduced to 0% and habituation started 20 min after reaching the 0% value. During anesthesia, the nose cone, cheek and neck supports were padded with a soft polyurethane foam layer (thickness = 4 mm). First, the head was positioned in the lower part of the nose cone, securing the upper incisors on the bite bar; then the upper part of the nose cone was attached by screws to the lower one and the anesthesia flow was switched from the snout mask to a tube feeding in the nose cone, in order to maintain the animal under anesthesia until head-fixation was completed. After moderately stretching the body of the animal, the neck support was screwed to the rat bed and its height was adjusted to match the anatomy of the rat. The cheek pads were gently tightened, and a single-loop 20-mm surface coil (Bruker Biospin, Ettlingen, Germany) was positioned on the head. Finally, the rear part of the body was further stabilized by taping it to the rat bed, whilst paying attention not to put pressure on the rat’s body.

After the conclusion of all habituation steps, the rats were brought to the MRI room for the last phase of the habituation protocol (MRI acquisition, Figure 1A). Here, a pneumatic breathing sensor (Model 1025T, Small Animal Instruments Inc., New York, NY, United States) was positioned under the animal’s abdomen to monitor the respiration rate. The scanner bed was warmed, and body temperature was monitored to achieve a sustained core temperature of ∼37*^o^*C. After head-fixation, the rat bed was placed in the MRI bore, lowering it until the foam layer of the animal’s neck padding touched the volume coil. Afterwards, imaging procedures were commenced.

### Stress Monitoring

In order to evaluate the wellbeing of the animals during the habituation and scanning procedures, different factors were monitored:

(1) fecal corticosterone metabolite (FCM) as an indicator of stress-related hormone levels;
(2) body weight, since rats exposed to restraint stress lose weight and do not return to their physiological weight for extended periods of time (Harris et al., 2006);
(3) respiration rate, because respiratory dysregulation is characteristic of stress or anxiety related behavior; and
(4) head motion levels, because stressed animals will try to escape from restraint. These were estimated during the imaging procedure.

#### Fecal Corticosterone Metabolite Assessment

Among the different stress-related hormones, corticosterone shows little variability across species, and represents the main stress-hormone for rodents (Joëls et al., 2018; Will et al., 2019). In order to avoid corticosterone fluctuations linked to the blood sampling procedure, non-invasive measurements have become established (Sheriff et al., 2011; Meyer and Hamel, 2014; Cinque et al., 2018). Fecal corticosterone metabolite (FCM) assessment is the most frequently used, since it has been widely validated and a tight relationship between circulating corticosterone and fecal corticosterone metabolites has been demonstrated (Cavigelli et al., 2005; Thanos et al., 2009; Cinque et al., 2018).

In this study, fecal samples were collected one week after the arrival of the animals in our facility, prior to any kind of manipulation (pre-handling), as well as 24 h and 72 h after each step of the habituation procedure. This was done to measure acute (24 h) and chronic (72 h) stress levels related to the corresponding habituation phase.

To collect fecal boli, each animal was gently removed from the home cage and was positioned in a single cage with clean bedding (polycarbonate rat cage type II, 430 × 290 × 201 mm). After the fecal sample was excreted it was collected and immediately stored at −20°C. At the end of the habituation period, each sample was prepared in triplicate for an enzyme immunoassay (EIA) according to the procedure described by the kit’s provider (KO14, Arbor Assays, Ann Arbor, MI, United States). The absorption was read with a multimode microplate reader (Varioskan LUX, Thermo Scientific, Schwerte, Germany) and analyzed employing a four-parameter logistic fit. Corticosterone concentrations were determined as ng/g of fecal matter. Acute and chronic FCM results were compared testing for significance using analysis of variance with repeated measures (rmANOVA), followed by a *post hoc* Fisher’s LSD test. Moreover, in order to compare each phase’s chronic FCM levels with the pre-handling FCM levels, six paired-two sample *t*-tests (Bonferroni correction for multiple comparisons) were conducted.

#### Functional Magnetic Resonance Imaging Motion Parameters Estimation

Estimated motion levels from fMRI data were obtained using MCFLIRT (Jenkinson et al., 2002), a tool from the FMRIB Software Library (Smith et al., 2004). No changes were made to the default parameters. Histograms of the absolute six motion parameters were computed in order to assess the extent of head motion (translations and rotations along the *X*, *Y*, and *Z* axes). The mean value of the absolute motion peaks estimated in all the subjects was also calculated, to evaluate the maximum motion in our datasets. The peaks for each single subject, for each axis and for each motion modality (translations and rotations) were detected extracting the absolute values of the local maxima and minima from the output MCFLIRT data. We defined the following criteria to identify motion peaks: in order to be detected as such, a peak must be larger than its two neighboring samples and have a prominence greater than or equal to the standard deviation of the specific subject, axis and motion modality. The mean was also extracted for the breathing rate values. Further motion quantification was performed to enable comparison of the values with data published in the scientific literature obtained from awake rat fMRI, computing the root mean square (RMS) and frame-wise displacement (FD) of head motion in the three dimensions (Power et al., 2012), as well as the mean standard deviation of the six time-courses.

### Magnetic Resonance Imaging and Functional Magnetic Resonance Imaging Procedures

The functional MRI (fMRI) sequence (based on a sparse temporal sampling technique) was designed to cover the whole brain – precisely from the cerebellum to the beginning of the olfactory bulbs – and optimized for low acoustic stress on the rats.

Imaging was performed using a small animal MRI system (7T horizontal bore, 70/30 USR, Bruker BioSpec, Germany). An 80 mm transmit/receive quadrature birdcage resonator was used for radio frequency transmission and a single-loop 20-mm surface coil for signal detection. ParaVision 6 was used to acquire the data. The animals were placed under isoflurane anesthesia (3% for the induction and 1.5% for the maintenance phase) for all the pre-fMRI phases that will be described in the following sections. The anesthesia was removed 20 min before starting the fMRI acquisition and reintroduced before the high-resolution anatomical scan (in order to be able to remove the animals from the fixation system at the end of the experimental session).

#### Pre-Functional Magnetic Resonance Imaging Procedures

At the beginning of each imaging session, a FLASH localizer was used to roughly assess the position of the animal’s brain, then images in three different and manually adjusted planes were acquired: first, the axial plane as, defined by the magnet reference frame was obtained. Then the adjusted horizontal plane, was acquired using the axial rat brain images as reference. Finally, the adjusted sagittal plane was obtained, using the horizontal rat brain images as reference. These planes were acquired one after the other using a multi slice RARE sequence with the following parameters: repetition time (TR) = 4000 ms, effective echo time (TEeff) = 40.37 ms, Rapid Acquisition with Relaxation Enhancement (RARE) factor = 8, no average, acquisition matrix = 256 × 128, field of view (FOV) = 32 × 32 mm, spatial resolution = 0.125 × 0.25 mm^2^, slice thickness = 1 mm, number of slices = 15 axial, 20 horizontal, and 17 sagittal; subsequently, after the measurement of the map shim, a localized shim of the area of interest (whole brain) was performed.

#### Functional Magnetic Resonance Imaging Acquisition

On the basis of the sagittal reference images, 21 coronal slices were positioned along the entire brain and acquired with single-shot multi-slice gradient-recalled echo Planar Imaging (GRE-EPI), optimized for low acoustic-noise-related animal stress (with sparse temporal sampling, not designed for high temporal resolution) with the following parameters. Echo time (TE): 15 ms, TR: 4000 ms, acquisition time (TA, time required to scan all the slices for each volume): 1500 ms, slice thickness: 1 mm, no slice gap, 4 FOV saturation slices, matrix size: 64 × 64, FOV: 32 × 32 mm^2^, Bandwidth: 250 kHz, 375 volumes. The total awake scan time was 25 min. The slice acquisition was not evenly spread over the 4000 ms of TR, but rather was packed in a TA of 1500 ms. While this slightly increases the sound pressure during the 1500 ms TA, the resulting long quiet period is expected to help keep the animals calm (Perrachione and Ghosh, 2013). This approach can also be used to deliver sound stimulation with low scanner-related noise contamination (Acoustic noise and functional magnetic resonance imaging: Current strategies and future prospects - Amaro et al., 2002 - Journal of Magnetic Resonance Imaging - Wiley Online Library).

#### High-Resolution Anatomical Imaging

At the end of the experimental session, high-resolution anatomical images, exactly aligned with the functional ones, were acquired using a RARE sequence with the following parameters: TR = 4000 ms, TEeff = 41 ms, RARE factor = 10, number of averages = 8, FOV = 32 × 32 mm^2^, 21 coronal slices, slice thickness 1 mm, no slice gap, matrix size = 256 × 256, spatial resolution = 0.16 × 0.16 mm^2^.

#### Resting-State Acquisition and Auditory Functional Magnetic Resonance Imaging Paradigm

On the last day of the habituation protocol (i.e., the MRI acquisition phase), 375 resting-state volumes were acquired in order to evaluate the motion levels. Two days later, when the habituation protocol was already concluded and validated, we tested the quality of our setup by applying basic acoustic stimulation that should produce a very predictable activation pattern.

The auditory fMRI experiment started with the acquisition of 20 initial resting-state volumes, followed by acoustic stimulation (335 volumes acquired). It ended with the acquisition of 20 final resting-state volumes.

Acoustic stimulation consisted of two kinds of sound blocks (Figure 1C), with a duration per block of 50 s (12 s of sound, 38 s inter-trial interval), each repeated fourteen times. One sound block comprised a melody (Mozart’s piano sonata K.448) and the other was scrambled version of the same melody generated by randomly concatenating 200–300 ms excerpts with a 30 ms linear cross-fade between excerpts. In this way, both the sound blocks had the same physical properties (pitch, loudness, frequencies), but the scrambled version lacked temporal coherence (melodic contour and rhythm). The sound blocks were emanated from two speakers (L010 ultrasound speaker, Kemo Electronic GmbH, Germany), positioned 20 cm in front of the animal’s head with an intensity of 70 dB. The repeated presentation of these two block types was completely randomized: The start of the acoustic presentation was synchronized with the start of volume acquisition, including a maximum programmed delay of ± 200ms.

### Functional Magnetic Resonance Imaging Data Analysis

#### Initial Preprocessing

Functional magnetic resonance imaging data were processed using the FMRIB Software Library (FSL) and custom-written MATLAB (Version 2018b, MathWorks, United States) functions. Data preprocessing comprised the following steps: export of the images in the Digital Imaging and Communications in Medicine (DICOM) format (done by the software ParaVision 6), conversion of the DICOM images into the Neuroimaging Informatics Technology Initiative (NIfTI) format using the Analysis of Functional NeuroImages (AFNI, version 18.1.32, Cox and Hyde, 1997) dcm2niix_afni function, upscaling the voxel size by a factor 10, and motion estimation and correction (MCFLIRT, Jenkinson et al., 2002). This was the last step for the datasets acquired during the “MRI acquisition chronic” phase of the habituation protocol (Figure 1A) since the only purpose here was to extract the motion parameters from the fMRI data.

For the auditory fMRI datasets, we continued the preprocessing through to non-brain tissue removal, by applying a manually drawn binary mask and spatial smoothing with a Gaussian kernel of 10 mm (upon upscaling). After these steps, the resulting preprocessed NIfTI file was split in single volumes and the motion parameter file generated by FSL during motion correction was converted in a format readable by SPM8^1^. The “art_global” graphical user interface (GUI), part of the MATLAB toolbox ArtRepair (Mazaika et al., 2007, 2009), was used to remove spikes in the time course by clipping all values higher than 3.04 standard deviations from a rolling mean. This value was calculated by feeding all the datasets to the toolbox, and was kept constant when de-spiking each dataset. The signal is measured relative to the global signal mean (mean time course computed over all the voxels) of the image. The volumes that are detected to be repaired are generated using linear interpolation from the nearest unrepaired volumes. After repair, the single volumes were merged into one “repaired” NIfTI file.

#### Data Denoising

As part of the preprocessing, the data were further processed to remove motion-related artifacts through independent component analysis (ICA) (Griffanti et al., 2014; Pruim et al., 2015). The criteria to label independent components (ICs) as noise are based on the correlation of the IC with the motion parameters calculated by FSL. More precisely, a general linear model (GLM), adopting the double-gamma hemodynamic response function (HRF) with a phase of 2 s, was generated by FSL. The auditory stimulation was modeled in order to verify that ICs that were removed did not substantially correlate with the stimulation model, in order to ensure that important information regarding auditory processes was not disregarded.

At this point, using the Multivariate Exploratory Linear Optimized Decomposition into Independent Components (MELODIC) GUI of FSL, ICA was run on the repaired data without any further preprocessing. In the post-statistics tab of the MELODIC GUI, the GLM model was provided. Moreover, the 6 motion parameters were used as explanatory variables (EVs). All the EVs were orthogonalized against each other. Two contrasts for each EV, one to detect positive correlation and the other for the negative correlation, were set, for a total of 6 EVs and 12 contrasts. The contrasts provided more detailed timeseries and allowed for the calculation of the correlation between the ICs and the EVs. The ICs were manually inspected according to well-established criteria (Moritz et al., 2003; Kelly et al., 2010; Robinson et al., 2013; Griffanti et al., 2017). In summary, ICs spatial maps, where at least 90% of the signal is localized in the brain periphery, with diffuse spotty patterns or large activation clusters covering at least 25% of the whole brain, with no regard for functional and anatomical boundaries, were marked as noise. ICs displaying at least 10% of activations in confined gray-matter clusters were labeled as signals. As a general rule, when there was doubt as to whether ICs represented noise or signals, they were labeled as signal ICs. The ICs were maked as noise if two or more of the secondary criteria described in literature applied, e.g. more than 50% of the Fourier frequency spectrum’s power lying above 0.1Hz, or large spikes, greater than five standard deviations, were found in the time-course. The repaired NIfTI was then denoised using FSL’s function “fsl_regfilt” to regress out of the fMRI data the components labeled as noise.

#### Blood-Oxygenation-Level Dependent Brain Activation

The last step of the single-subject processing comprised running the FMRI Expert Analysis Tool (FEAT) (Woolrich et al., 2001), in order to apply a high-pass temporal filter (cutoff at 100 s) and calculate the activation maps. The GLM for the statistics was set as in the pre-ICA step. The motion parameters were not included as EVs, since motion-related ICs had already been linearly regressed out. In total 2 EVs and 4 contrasts were set, without orthogonalizing the EVs. The temporal derivative was added to the double gamma HRF with a phase value of 2 s – in order to compensate for delays in the hemodynamic response to neural activity – and the high-pass temporal filtering was applied to the model. The selection of the double-gamma HRF with a phase of 2 s was based on recent findings by others who reported a faster progression of the rat cortical HRF compared to the human HRF, as well as pilot studies that were conducted to visually inspect the best general HRF fit (Peng et al., 2019; Lambers et al., 2020).

#### Registration to a Template

Before proceeding with the group analysis, each subject was registered (linear interpolation, 12 degrees of freedom) to a common high-resolution MRI anatomical template obtained by registering all the single subject high-resolution anatomical images with each other and averaging them using FSL’s FLIRT. For the group results’ visualization, the activation maps were linearly co-registered to the SIGMA rat MRI brain template (Barrière et al., 2019).

#### Group Analysis

In order to detect the statistically significant activated brain regions, the single subject final results were fed to FEAT and the mixed-effect model (FLAME1 + 2) was selected. After registration, the *Z* statistics images were obtained setting a cluster-threshold (to check for clusters continuity) with a minimum *Z*-value of 3.1 (corresponding to *p* < 0.001) and each cluster’s estimated significance level was compared with the cluster probability threshold (*p* = 0.05) in order to generate the final statistical map.

#### Regions of Interest Analysis

**FIGURE 2:**
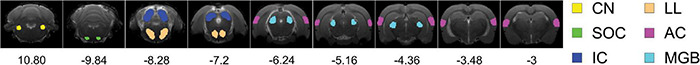
Regions of interest (ROIs) used in the blood-oxygenation-level dependent (BOLD) percent signal change computation. Six Regions of interest (ROIs) were manually drawn using a rat brain atlas as reference. The BOLD percent signal change was calculated for these regions. The distance from bregma is displayed below each anatomical magnetic resonance imaging (MRI) brain slice.

Regions of interest (ROIs) were manually drawn on the MRI anatomical template according to a rat brain atlas (Paxinos and Watson, 2007). The 6 ROIs chosen comprised the cochlear nucleus (CN), superior olivary complex (SOC), inferior colliculus (IC), lateral lemniscus (LL), medial geniculate body (MGB) and auditory cortex (AC) (Figure 2). After rescaling the ROIs to the single subjects’ functional space, FSL’s “featquery” was used to extract the BOLD percent signal change for the ROIs for each single subject and the group average and standard deviation were computed with a custom-written MATLAB code.

## Results

### Incremental Habituation to Head-Restraint and Functional Magnetic Resonance Imaging Scanning Results in Restoration of Corticosterone to Pre-handling Levels While Not Affecting the Weight of the Animals

Animals were habituated over a period of three weeks and eighteen separate sessions to the awake restraint conditions (Figure 1A). Corticosterone levels (Supplementary Table 2) were monitored by analyzing fecal corticosterone metabolite (FCM) concentrations of the stress hormone. An rmANOVA indicated a significant main effect of habituation on the FCM levels between the acute and chronic habituation phases (*F*(1,5) = 66.91, *p* < 0.001, η_*p*_^2^ = 0.93, *n* = 6). A *post hoc* test indicated that animals’ stress, from the acute to the chronic phase, decreased after habituation to the body restraint, darkness, and head fixation chronic conditions (*p* < 0.001).

In addition, prior to the beginning of the habituation, the FCM concentration was 71 ± 24 ng/g (Figure 1B). This value was used as a reference value to evaluate the effect of different habituation steps on the FCM level. Six paired-two sample two-sided t-tests (Bonferroni correction for multiple comparisons) were used to make a comparison between the chronic phase of different habituation steps and the pre-handling level of the FCM. The results revealed that the FCM levels after each habituation step were lower or equal to pre-handling levels (Figure 1B). The FCM levels during the chronic phase of body restraint, darkness, MRI noise and MRI acquisition steps were significantly lower than the pre-handling level (*t*(5) ≥ 5.25, *p* < 0.03, *n* = 6). There was no significant difference between the chronic phase of handling and head fixation steps with the pre-handling level of the FCM (*t*(5) ≤ 3.90, *p* > 0.07, *n* = 6).

Moreover, daily monitoring of the weight of the animals revealed no evidence of weight loss throughout the entirety of the habituation and recording phases (Supplementary Table 1). Taken together, these findings indicate that this gradual habituation procedure is effective in acclimatizing the animals to the experimental conditions and habituate them to each phase over a period of three days. To verify this interpretation we went on to assess key imaging parameters such as motion and the reliability of fMRI recordings.

### Low Estimated Motion Parameters Are Accompanied by Stable Breathing During the Awake Functional Magnetic Resonance Imaging Scans and Consistent Brain Activation Patterns

**FIGURE 3:**
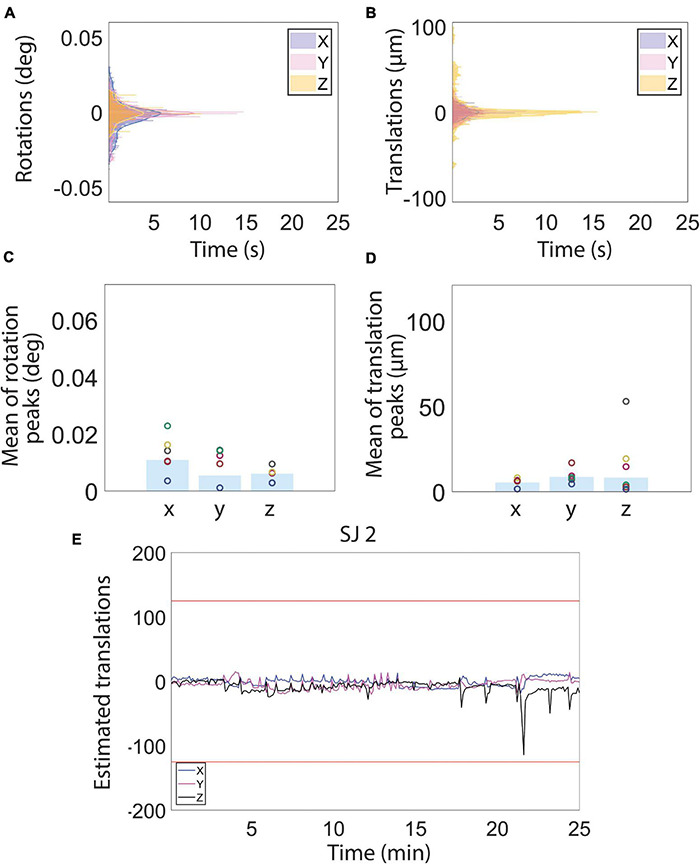
Motion estimation derived from magnetic resonance images during resting state functional magnetic resonance imaging (fMRI) acquisition. **(A–D)** Histograms **(A,B)** and bar charts showing the peak mean of the estimated rotation **(C)** and translation **(D)** motion parameters. **(E)** Examples of estimated translation levels for a single subject (SJ 2). The red lines represent 1/4 of the voxel size. (See Figure 4 for motion traces of all other subjects). Low estimated translation values (smaller than 1/4 of voxel size) during the whole fMRI acquisition reflect a normal physiological state of the subjects, indicative, in turn, of low stress levels.

**FIGURE 4:**
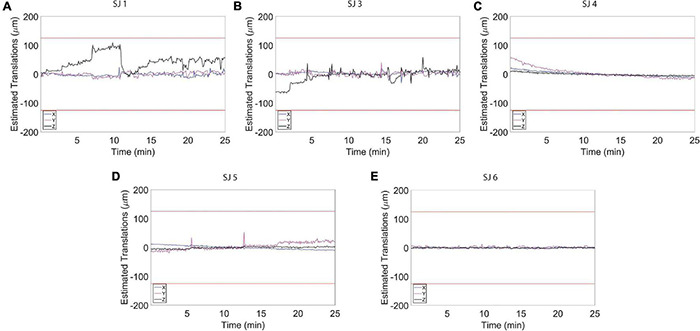
Estimated motion levels for individual animals during the resting state functional magnetic resonance imaging (fMRI) acquisition. **(A–E)** Estimated motion levels (translations) for five individual animals. Low estimated motion parameter values, evident during the whole fMRI acquisition for all subjects, reflect a physiological and calm state of the habituated animals. The red lines represent 1/4 of the voxel size.

A total of 375 resting-state volumes were acquired in order to evaluate the motion levels. The motion traces and the translation and rotation assessments (Figures 3A,B) revealed that the animals typically moved very little during image acquisition. On the few occasions during which the animals moved, the motion was of small amplitude. In particular, the average peaks of head motion rotations were always lower than 0.015° (Figure 3C) and the average peaks of head motion translations were always lower than 20 μm (Figure 3D). The maximum translation peak was of 109.86 μm (along the *Z* axis) and it was registered in rat subject number two (SJ2). Motion estimation traces were never higher than 1/4 of the voxel size for all the subjects (Figures 3E, 4), which when compared to literature, corresponds to levels that permit effective and accurate fMRI recordings (Lahti et al., 1998, 1999). Moreover, the mean (± S.E.M.) of the standard deviation was 5.8 ± 0.9 μm for the translations along *X*, 9.3 ± 2.2 μm for the translations along *Y*, and 11.4 ± 4.3 μm for the translations along *Z*. The mean FD (± S.E.M.) was 11.6 ± 0.4 μm and the mean RMS (± S.E.M.) was 0.9 ± 0.05 μm.

In addition, the respiration rate was constantly monitored during the awake fMRI scans. The respiration rate for all animals was in the range of 85 ± 7 breaths/min, which is in the physiological range for rats (Hofstetter et al., 2006).

Taken together, these findings show that it is possible to achieve low motion levels accompanied by very few spikes and a general stable animal physiology with the proposed approach.

### Imaging of Auditory Information Processing Using Awake Functional Magnetic Resonance Imaging

**FIGURE 5:**
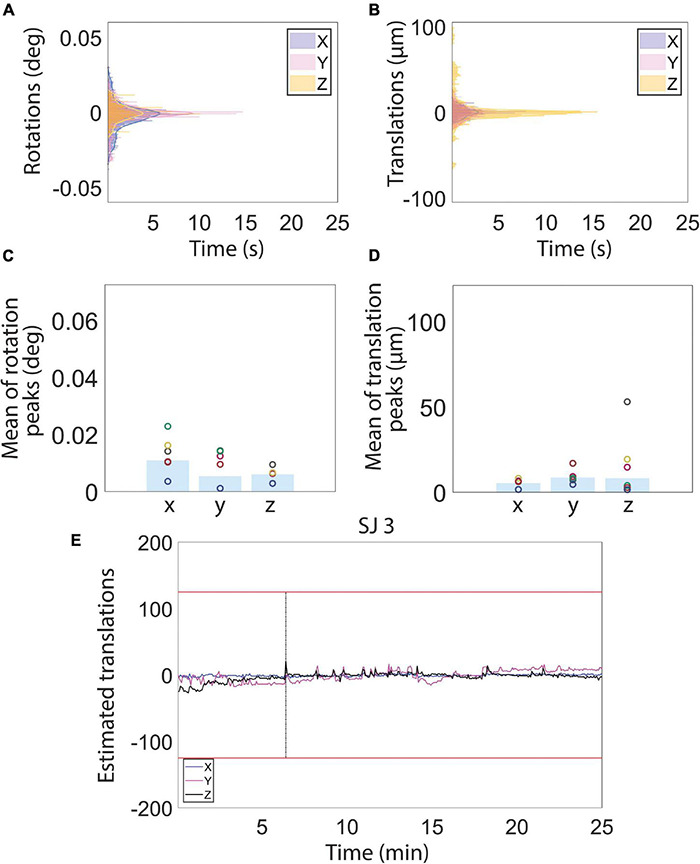
Motion estimations derived from MR images during the auditory functional magnetic resonance imaging (fMRI) acquisition. **(A–D)** Histograms **(A,B)** and peak mean of the estimated rotation **(C)** and translation **(D)** motion parameters. **(E)** Representative example of estimated translation levels for a single subject (SJ 3). (See Figure 6 for motion traces of all other subjects). The red lines represent 1/4 of the voxel size. The vertical black line indicates the location of a spike in the time course that was removed using the Matlab toolbox, ArtRepair. Low estimated motion parameter values recorded during the whole fMRI acquisition reflect a physiological and calm state of the animals.

**FIGURE 6:**
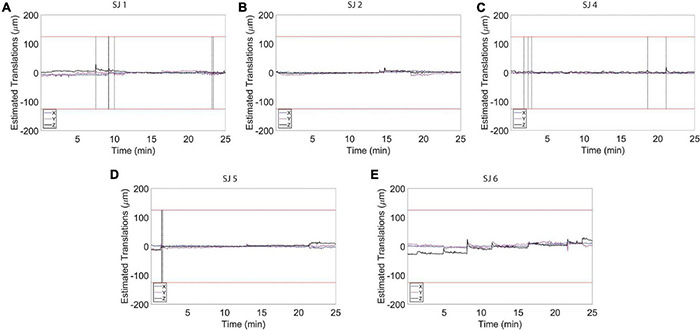
Estimated motion levels for individual animals during auditory functional magnetic resonance imaging (fMRI) acquisition. **(A–E)** Estimated motion levels (translations) for five individual animals during auditory fMRI acquisition. Motion levels during the auditory task are lower and more stable compared to the levels detected during the resting state acquisitions (Figure 4). The red lines represent 1/4 of the voxel size. The vertical black line indicates the location of a spike in the time course that was removed using the Matlab toolbox, ArtRepair.

To assess to what extent our habituation protocol can improve signal resolution of auditory information processing, we exposed the animals to auditory stimulation (Figure 1C). The test sound block used was expected to produce a basic and predictable activation pattern that allowed us to assess the sensitivity and stability of our imaging strategy. During the whole scanning session, the breathing rate was stable at 82 ± 6 breaths/min. Also in this case, the histograms for the fMRI volume rotations (Figure 5A) and for the fMRI volume translations (Figure 5B) show that the animals were very calm during the fMRI experiment. The average peaks of head motion rotations were always lower than 0.005° (Figure 5C) and the average peaks of head-motion translations were always lower than 7 μm (Figure 5D). The maximum translation peak was of 30.63 μm (along the *Z* axis) in SJ1. In general, motion estimation traces were never higher than 1/4 of the voxel size for all the subjects (Figures 5E, 6).

**FIGURE 7:**
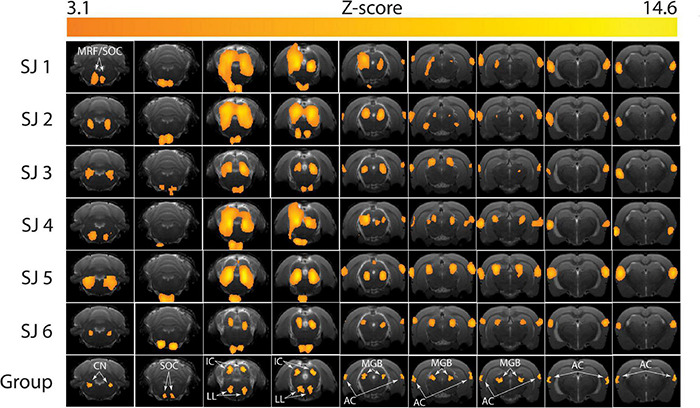
Brain activation patterns during the functional magnetic resonance imaging (fMRI) auditory task. The *Z*-score maps are reported for each subject (SJ 1-6) at the end of the first-level analysis, and for the whole group after the higher-level analysis (bottom row). MRF, medullary reticular formation; CN, cochlear nucleus; SOC, superior olivary complex; IC, inferior colliculus; LL, lateral lemniscus; MGB, medial geniculate body; AC, auditory cortex. Higher single-subject-consistency is present in the IC, LL, MGB, and AC and lower single-subject-consistency in the CN and SOC.

The brain activation pattern was similar in all the six subjects (Figure 7). In particular, no activation was observed in slices 1 to 4 (covering the cerebellum), as well as in slices 14-21 (ranging from the rostral part of the dorsal hippocampus to the olfactory bulbs). Four animals (SJ 1, 2, 4, 5) showed activations in the medullary reticular formation (MRF), whereas two animals did not exhibit activation in this region (SJ 3, 6). In the apical brain region (slices 9-11, Figure 7) we detected some inter-subject variability, when comparing the activated regions: the auditory cortex and the medial geniculate body were not always bilaterally activated. The group analysis (Figure 7, “Group”) revealed statistically significant activated regions (*Z*-score threshold for cluster estimation: 3.1, p = 0.05 as the cluster probability threshold). These comprised the cochlear nucleus (CN), the superior olivary complex (SOC), the inferior colliculus (IC), the lateral lemniscus (LL), the medial geniculate body (MGB) and the auditory cortex (AC). In this case, a very typical pattern of auditory activations emerged. It exactly covered the auditory brain regions, and the MRF activation is not significant from the group analysis. We believe that this activation could be related to an overlap with the SOC activation, since they were detected as a single cluster from FSL. The BOLD percent signal change was calculated for the aforementioned regions and the results are summarized in Table 1.

**TABLE 1:** Blood oxygen level-dependent (BOLD) percent signal change in the auditory regions of interest (ROIs).

ROI	Number of voxels	BOLD %
Cochlear Nucleus	21 ± 3	2.5 ± 1.2%
Superior Olivary Complex	21 ± 4	4.9 ± 2.0%
Inferior Colliculus	133 ± 12	3.0 ± 0.6%
Lateral Lemniscus	35 ± 2	2.5 ± 1.0%
Medial Geniculate Body	39 ± 3	1.4 ± 0.3%
Auditory Cortex	72 ± 8	1.7 ± 0.5%

*The percent blood oxygen level-dependent (BOLD) signal change from baseline was computed (using FSL) for the six auditory regions of interest (ROIs). The group average ± standard deviation (n = 6) are shown, the average (± standard deviation) number of voxels included in each ROI for the six subjects is also reported.*

Taken together, these findings show how awake fMRI-habituation of rats to achieve stress levels that are not different from pre-handling levels, low motion estimates, stable physiological parameters and the use of a sparse imaging paradigm, allow for sensitive detection of brain activity linked to auditory information processing.

## Discussion

In this study, we describe a novel habituation and data acquisition approach for fMRI in awake rodents that minimizes stress levels, motion artifacts and allows discrimination of auditory brain structures, despite acoustic scanner noise. BOLD signals detected in the thalamus and reticular formation indicate that animals directed their attention to the acoustic stimuli, consistent with effective implementation of acoustic stimulation, despite the backdrop of scanner noise. We implemented a gradual acclimatization of adult rats to individual scanning stressors, spanning a 3-week training period, using a low-cost and easy to build animal head-fixation system that does not involve the surgical implementation of an anchoring socket on the animal’s head. Furthermore, we tailored our gradient echo EPI acquisition so as to lower the stress of the animals during fMRI, thereby achieving an effective compromise with regard to the temporal and spatial resolution of BOLD images.

Since stress can distort cognitive responses, creating a bias in the collected data (Diamond et al., 1992; Kim and Diamond, 2002; Joëls et al., 2018), we validated our adapted habituation protocol by assessing the animals’ stress levels during the eighteen-session habituation protocol. Corticosterone and fecal corticosterone metabolite (FCM) levels provide reliable insight into stress levels in rodents (Cinque et al., 2018; Joëls, 2018). Furthermore, state-dependent fluctuations in corticosterone levels exert very specific impacts on cognitive performance (Hurtubise and Howland, 2017). In addition, a U-shaped relationship between corticosterone levels and spatial memory efficacy has been described in rodents (Park et al., 2006) suggesting that elevating corticosterone levels into the stress domain impairs the function of key memory structures such as the hippocampus (Diamond et al., 1992; Park et al., 2008). For these reasons, we measured FCM levels as an indicator of the stress of the animals. Our assessment revealed non-significantly different FCM levels between the pre-handling and the MRI acquisition conditions. Thus, we could show that the gradual habituation to the awake fMRI conditions resulted in non-stressed animals. The strongest stressor during habituation comprised *de novo* head-fixation, but repeating the habituation to this condition for three consecutive days resulted in a significant reduction of corticosterone levels back to pre-handling levels. This finding emphasizes the important of repeated habituation to head fixation. The significantly lower than pre-handling FCM levels are puzzling: low levels of circulating corticosterone are not optimal for cognition (Diamond et al., 1992), but FCM levels are not sufficiently sensitive to reveal minor stress, or acute stress, of short duration (Siswanto et al., 2008). Therefore, these lower FCM levels should not be interpreted in the same way as low circulating corticosterone levels. Low FCM levels simply reflect a non-stressed status of the animals, or that the stressor was not potent enough to be detected in feces.

As an additional stress indicator, breathing rate was constantly monitored during the whole fMRI experimental procedure. The respiration was stable at 82 ± 6 breaths/min, which reflects a physiological and calm breathing rate for rats (Hofstetter et al., 2006). The fact that the animals were stable in their breathing rate, without presenting stress-associated values, is a further indicator of their physiological state. Body weight did not decline throughout the habituation and recording phases. Taken together, the stable breathing rate, constant body weight and the low motion levels during the fMRI acquisition reflect that the animals were effectively habituated to the awake fMRI experiments.

On the last day of the habituation protocol, a resting-state fMRI sequence was acquired in order to assess the motion levels and further evaluate the animals’ behavior. Motion estimation traces were never higher than 1/4 of the voxel size for all the subjects, which compared to reports by others, correspond to levels that permit effective and accurate fMRI recordings (Lahti et al., 1998, 1999). The effects of head-motion are particularly complex and not yet fully understood. BOLD percent signal changes are usually of small amplitude. Furthermore, head, or body, motion will affect fMRI images through direct head displacements, or induced fluctuations of magnetic inhomogeneities in the brain, along with spin-history effects (Friston et al., 1996; Power et al., 2015; Xifra-Porxas et al., 2020). Moreover, motion can temporally correlate with the task presentation making the discrimination of brain activity from motion artifacts even more complicated. This results in a compromised interpretation of the fMRI data (Johnstone et al., 2006). To circumvent these problems two possible approaches are possible: it is possible to use nuisance regressors to regress out motion artifacts (Caballero-Gaudes and Reynolds, 2017), and it is possible to adopt strategies to minimize the motion already during the data acquisition phase. Pursuing the latter strategy was the focus of this work.

In this study we report how gradual habituation, combined with a sparse temporal sampling sequence, results in very low motion and stress levels in habituated young adult rats during fMRI acquisition. In this regard, several important pioneering studies dedicated to awake fMRI in rats have already shown how a relatively short (7-10 day) habituation procedure can lead to circulating corticosterone levels that do not differ from baseline ones (King et al., 2005; Febo, 2011; Chang et al., 2016; Ferenczi et al., 2016; Ma et al., 2017, 2020). Indeed, in both the present and previously published studies (Table 2), stress levels returned to baseline levels after the habituation procedure. Nonetheless, motion levels remained relatively high in those published studies and could have created a confound in accurate data acquisition. This is noteworthy because it indicates that sole use of plasma corticosterone, or FCM levels, as a biomarker for stress habituation is insufficient. Comparing the published motion levels in awake rats with the ones we obtained in the present study, we conclude that our protocol (that includes gradual habituation) led to lower motion estimates. More specifically, we report here a mean (± S.E.M.) standard deviation of translations of 5.8 ± 0.9 μm for the *X* axis and 9.3 ± 2.2 μm for the *Y* axis, that are respectively around 8- and 16-fold smaller than the values reported by King et al. (2005). The mean motion root-mean-square (± S.E.M.) is 0.9 ± 0.05 μm, was thus around 72 times smaller than values reported by Ferenczi et al. (2016) and 30 times smaller than values reported by Chang et al. (2016). The mean frame-wise displacement (± S.E.M.) in our study is 11.6 ± 0.4 μm, which is around 13 times smaller than what was reported in literature (Ma et al., 2017) and 4 times smaller than the value of 50 μm reported by Ma et al. (2020). To the best of our knowledge, we report here the lowest motion levels during awake rat fMRI in the current literature. We believe that this is one important reason why very detailed whole brain auditory blood-oxygen-level-dependent imaging (BOLD) fMRI patterns were detectable at the single subject and group levels (Figure 7). Keeping in mind that habituation must have multiple goals, including a reduction of reducing stress levels as well as having quiet and minimally moving animals, we recommend that in addition to stress level monitoring, head motion estimates, breathing level and weight assessments should be used as biomarkers for effective habituation to an awake fMRI paradigm in rodents.

**TABLE 2:** Comparison of the currently available awake-rat-functional magnetic resonance imaging (fMRI) strategies.

Studys	Rat strain	Rat age	Duration and type of habituation	Corticosterone levels	Respiration rate	Type of MRI study	Motion Levels
Present study	Male Lister Hooded	4 weeks	Gradually introduced rats to the experimental conditions, 3 weeks 20–60 min/day	FCM levels return to pre-handling values after habituation	85 ± 7 breaths/min on last habituation day	Task-based auditory fMRI	Mean (± S.E.M.) SD of translations: 5.8 ± 0.9 μm (*X*), 9.3 ± 2.2 μm (*Y*). Mean (± S.E.M.) RMS: 0.9 ± 0.05 μm. Mean (± S.E.M.) FD: 11.6 ± 0.4 μm
King et al., 2005	Male Sprague Dawley	/	Rats exposed to the final experimental conditions, 8 days, 90 min/day	Plasma corticosterone levels significantly decreased on day 5 and 8 compared to day 1 of habituation.	∼95 ± ∼7.5 breaths/min on day 8	Resting state fMRI	Mean (± S.E.M.) SD of translations: ∼60 ± ∼50 μm (*X*), ∼150 ± ∼40 μm (*Y*).
Febo, 2011	Male Sprague Dawley	/	Rats exposed to the final experimental conditions, 5 days, 20–50 min/day	/	/	stimulus fMRI	/
Chang et al., 2016	Male Sprague Dawley	Adult	Rats exposed to the final experimental conditions, 8–10 days, 30 min/day	Plasma corticosterone levels showed a trend toward decreasing on day 8 of the habituation, but increased significantly during the fMRI scan.	73.9 ± 6.1 breaths/min during fMRI experiments	Resting state and stimulus (air puff) fMRI	/
Ferenczi et al., 2016	Female Long-Evans TH-Cre rats and Wild-type male Sprague Dawley rats	/	Rats exposed to the final experimental conditions, 5–10 days, 5–45 min/day	/	> 100 breaths/min during fMRI experiments	Optogenetic fMRI	Mean (± S.E.M.) RMS: 65 ± 3 μm.
Ma et al., 2017, 2020	Male Long-Evans	Adult	Rats exposed to the final experimental conditions, 7 days	/	∼ 82 breaths/min	Resting state fMRI	Mean (± S.E.M.) FD: 150 ± 20 μm (2017), 50 ± 3 μm (2020)
Stenroos et al., 2018	Male Wistar	Adult	Rats exposed to the final experimental conditions, 4–9 days, 15–45 min/day	Plasma corticosterone levels return to baseline by day 5 of habituation.	∼ 160 breaths/min	Resting state fMRI	/

*In this table the habituation strategy presented in the current work (“present study”) is compared with other published strategies. With the exception of our study, none of the strategies used adapted a gradual habituation of the animals to the awake fMRI condition, rather the animals were habituated to all aspects of the final awake condition over a period of days. Differences in stress (reflected by corticosterone levels) and motion levels (far right column) are reported where available, as indicators of the final habituation outcome.*

Further studies would be needed to disambiguate if the more gradual habituation procedure, or the use of a sparse imaging protocol, or the adaptation of rats at a young age, or a combination of some, or indeed all of these conditions favor low-motion imaging. Nonetheless, we believe that our approach enables an effective reduction of motion estimates under conditions of relatively mild stress levels. As a consequence, the possibility that higher-level and cognitive brain areas are affected is lower compared to previous studies, thereby improving the reliability and reinforcing the interpretation of the experimental output.

Having established a procedure that enables the acquisition of accurate BOLD measurements, we assessed cortical responses to the presentation of acoustic stimulation to our rats. We noticed interesting differences in the motion traces when the rs-fMRI acquisition and the auditory fMRI acquisition were compared: the motion traces during the auditory fMRI experiments were more stable and exhibited lower amplitude motion-spikes compared to the responses detected during rs-fMRI. This might be related to the saliency and novelty of the auditory stimuli, which corresponded to periods comprising no stimulation (only the scanner noise was present), with periods of active auditory stimulation.

It is noteworthy that MRI noise is typically highly problematical when conducting auditory fMRI studies. Small animal fMRI usually takes advantage of high-resolution imaging, which relies on the rapid switching of strong magnetic gradients with short gradient rise times. A natural consequence of this procedure is the generation of high-intensity acoustical noise, that is a stressor for the animals. This can potentially affect the well-being of the awake animals and can affect auditory fMRI studies. In addition, fMRI acoustic noise activates auditory regions and can reduce the responsiveness of auditory structures to the actual auditory stimuli. Taken together, this reduces and modulates the measurable relative BOLD signal (stimulus against baseline) and, obviously, generates combined auditory responses, thereby confounding data acquisition from the whole experiment. There are multiple approaches that can be used to reduce the effects of MRI-generated acoustical noise. One of them comprises use of sparse imaging paradigms. Moreover, we believe that the use of a sparse imaging sequence is therefore another important factor, in synergy with the habituation protocol, that contributed to the consistent BOLD patterns that we recorded during experiments. Sparse imaging elicits more robust responses in the auditory cortex when compared to other approaches, such as continuous imaging that brings confounds for auditory studies (Petkov et al., 2009). Although sparse imaging paradigms have some limitations – such as a decreased temporal resolution of the hemodynamic response and slower acquisition of consecutive volumes (Schwarzbauer et al., 2006) – it offers the opportunity to apply stimuli and to sample hemodynamic alterations, without artifacts due to the continuous scanner gradient switching (Nebel et al., 2005). One major advantage is that the sparse imaging paradigm (70 dB noise 1.5 s duration repeated every 4 s for 25 min) reduces the noise exposure of the animals. Prolonged (> 2 week) white noise exposure at 70 dB leads to hyperacusis in rodents (Thomas et al., 2019). In non-auditory fMRI studies, the use of earplugs can be considered to further reduce noise exposure. All in all, designing a sparse imaging sequence allowed us to achieve two goals at the same time: to reduce *a priori* the animals’ stress that could originate from the continuous scanning noise, and to obtain robust responses across the whole auditory pathway.

The whole-brain analysis results obtained in the acoustic stimulation experiment revealed that the activated cortical structures corresponded to those that are part of the central auditory pathway. In summary, when the sound waves reach the ear canals, they are guided into the inner ear where they encounter the cochlea and its spiral organ of Corti that converts the motion of its hair cells into electrical signals. These electrical inputs are transmitted to the cochlear nucleus (CN), from which they diverge into several parallel ascending tracts. From the dorsal CN, most fibers ascend in the contralateral lateral lemniscus (LL); other fibers ascend in the ipsilateral LL. The fibers from the ventral CN project to the contralateral superior olivary complex (SOC), which in turn projects upwards through the lateral lemniscus. The ascending auditory tracts converge now in the inferior colliculus (IC), which is the principal source of input to the medial geniculate body (MGB) and auditory cortex (AC) (Malmierca, 2003). Our fMRI experiment lacks the temporal resolution needed to study sequential auditory activation, but the fact that all the expected brain regions were activated by the auditory stimulus provides further evidence that our fMRI approach enables the investigation of functional auditory activation in awake rats.

Most importantly, our experiments show high reproducibility, since the detected brain activation pattern was consistent in every single animal tested, with higher single-subject-consistency in the IC, LL, MGB, and AC and lower single-subject-consistency in the CN and SOC. The group analysis then confirmed the expected activations across the whole auditory pathway. Special attention in the evaluation of the outcome of the habituation procedure should be given to the higher-level auditory structures – such as the auditory cortex (AC) and the medial geniculate body (MGB). It has been reported by others that the activation in higher-level auditory brain structures is not very robust and simple to detect (Cheung et al., 2012; Blazquez Freches et al., 2018). This may be due to local differences in vasculature, neurovascular coupling, energy consumption, or neuronal activity (Logothetis and Wandell, 2004; Suta et al., 2008; Cheung et al., 2012). Other factors that can alter and suppress the BOLD signal in these regions are the use of anesthesia – which has a stronger effect on the AC than on the IC or other lower-level structures (Cheung et al., 2001; Ter-Mikaelian et al., 2007; Schumacher et al., 2011) – high motion in awake animals and the low sound pressure level of the stimulation (Gao et al., 2015). While in several studies the sound stimulation intensity reached up to 90 dB (Gao et al., 2015; Lau et al., 2015; Blazquez Freches et al., 2018; Chen et al., 2020), the present study is the first one in which BOLD activation is detected in lower- and higher-level brain auditory regions in awake rats with a sound intensity as low as 70 dB.

The abovementioned reduction in motion artifacts during auditory stimulation may have resulted from changes in attention in the animals during the presentation of the sound blocks. BOLD signal elevations in the thalamus and reticular formation are consistent with focused attention. Whereas the thalamus, and most especially the reticular thalamus supports selective attention, especially to sensory information (Guillery et al., 1998; Phillips et al., 2021), the reticular formation is critically involved in directing cortical arousal and attention to salient stimuli (Jones, 2003). The detection of the activation of these structures during melodic acoustic stimulation supports that our gradual habituation strategy to awake fMRI supports the discrimination of cognitive processes in the absence of stress confounds.

## Conclusion

In this study, we described an adapted habituation protocol aimed to minimize stress and motion levels during wake head-restrained fMRI in rodents. While in previous studies the animals were habituated using a more direct approach (King et al., 2005; Tsurugizawa et al., 2012; Chang et al., 2016; Stenroos et al., 2018), the goal of this study was to gradually and incrementally introduce the animals to the scanning set-up and procedure. The approach was validated based on FCM, breathing, motion and BOLD levels. Specifically, the consistency in the BOLD activation pattern obtained after habituation reflected the overall stability and sensitivity of our platform. Thus, we demonstrated that our habituation protocol results in very stable breathing and minimal motion levels, leading to robust discrimination of brain activation. Functional validation occurred by means of the presentation of acoustic stimulation in the form of discriminable and defined auditory wavelengths and patterns. We not only effectively detected auditory structure that would be expected to relay and process this information, but also detected the involvement of the thalamus and reticular formation during melody presentation, despite the backdrop of scanner noise.

In conclusion, habituation strategies that normalize the stress response to benign levels and minimize motion during head-restraint allow for high-quality fMRI data acquisition, avoiding cognition- and motion-related data biases. We, therefore, believe that these strategies will expand the possibilities of neuroscientific research using fMRI, by circumventing the limitation of studies to sedated animals, or to resting state analysis in awake rats. This approach can therefore open up new opportunities to study a broad spectrum of brain functions in rodents that, in turn, can be compared and translated to results obtained in human subjects. This is fundamental both in terms of acquiring a better understanding of brain function, and paving the way for functionally meaningful assessments of therapeutical strategies at the mechanistic level in rodents.

## Data Availability Statement

The original contributions presented in the study are included in the article/Supplementary Material, further inquiries can be directed to the corresponding author/s.

## Ethics Statement

The animal study was reviewed and approved by the Ethics Committee of the federal state of NorthRhine Westphalia (NRW) (Landesamt für Naturschutz, Umweltschultz und Verbraucherschutz (LANUV), Bezirksamt Arnsberg.

## Author Contributions

DM-V and GR designed the study and wrote the manuscript that was edited by all authors. GR conducted the experiments. XH and MB developed the MRI analytical approach. All authors analyzed the data.

## Conflict of Interest

The authors declare that the research was conducted in the absence of any commercial or financial relationships that could be construed as a potential conflict of interest.

## Publisher’s Note

All claims expressed in this article are solely those of the authors and do not necessarily represent those of their affiliated organizations, or those of the publisher, the editors and the reviewers. Any product that may be evaluated in this article, or claim that may be made by its manufacturer, is not guaranteed or endorsed by the publisher.

## Footnotes

1
www.fil.ion.ucl.ac.uk/spm/
