# Supplementary material for: Gradual Restraint Habituation for Awake Functional Magnetic Resonance Imaging Combined With a Sparse Imaging Paradigm Reduces Motion Artifacts and Stress Levels in Rodents
Source: Front Neurosci. 2021 Dec 21;15:805679. doi: 10.3389/fnins.2021.805679 (PMC8724036; doi:10.3389/fnins.2021.805679)
Supplement: Supplementary file 1 [file Table_1.pdf]

Habituation phase	Rat	Weight (g)	Mean (g) ±S.E.M.
Pre-handling	SJ1	143	138±5
	SJ2	122	
	SJ3	158	
	SJ4	144	
	SJ5	130	
	SJ6	129	
Handling Day1	SJ1	143	138±5
	SJ2	121	
	SJ3	159	
	SJ4	144	
	SJ5	133	
	SJ6	129	
Handling Day2	SJ1	145	141±5
	SJ2	124	
	SJ3	161	
	SJ4	146	
	SJ5	134	
	SJ6	133	
Handling Day3	SJ1	151	145±5
	SJ2	129	
	SJ3	164	
	SJ4	151	
	SJ5	139	
	SJ6	137	
Body restraint Day1	SJ1	165	159±6
	SJ2	140	
	SJ3	182	
	SJ4	160	
	SJ5	151	
	SJ6	156	
Body restraint Day2	SJ1	168	162±6
	SJ2	142	
	SJ3	185	
	SJ4	166	
	SJ5	153	
	SJ6	158	
Body restraint Day3	SJ1	176	169±6
	SJ2	151	
	SJ3	191	
	SJ4	174	
	SJ5	156	
	SJ6	163	
Darkness Day1	SJ1	197	185±6
	SJ2	171	
	SJ3	209	
	SJ4	183	
	SJ5	172	
	SJ6	175	
Darkness Day2	SJ1	198	186±6
	SJ2	171	
	SJ3	211	
	SJ4	185	
	SJ5	175	
	SJ6	175	
Darkness Day3	SJ1	198	188±6
	SJ2	175	
	SJ3	212	
	SJ4	187	
	SJ5	178	
	SJ6	177	
Habituation	Rat	Weight	Mean (g)
phase		(g)	±S.E.M.
MRI noise Day1	SJ1	203	196±5
	SJ2	183	
	SJ3	218	
	SJ4	199	
	SJ5	184	
	SJ6	190	
MRI noise Day2	SJ1	205	198±6
	SJ2	184	
	SJ3	220	
	SJ4	200	
	SJ5	184	
	SJ6	192	
MRI noise Day3	SJ1	208	200±6
	SJ2	188	
	SJ3	223	
	SJ4	201	
	SJ5	186	
	SJ6	194	
Head fixation Day1	SJ1	220	212±5
	SJ2	198	
	SJ3	230	
	SJ4	215	
	SJ5	201	
	SJ6	210	
Head fixation Day2	SJ1	224	214±5
	SJ2	200	
	SJ3	231	
	SJ4	217	
	SJ5	202	
	SJ6	210	
Head fixation Day3	SJ1	226	215±5
	SJ2	202	
	SJ3	231	
	SJ4	218	
	SJ5	203	
	SJ6	212	
MRI acquisition Day1	SJ1	231	220±5
	SJ2	206	
	SJ3	234	
	SJ4	226	
	SJ5	209	
	SJ6	216	
MRI acquisition Day2	SJ1	234	224±4
	SJ2	211	
	SJ3	238	
	SJ4	229	
	SJ5	215	
	SJ6	218	
MRI acquisition Day3	SJ1	236	227±4
	SJ2	215	
	SJ3	240	
	SJ4	232	
	SJ5	218	
	SJ6	220	
Auditory task	SJ1	249	239±5
	SJ2	223	
	SJ3	255	
	SJ4	241	
	SJ5	230	
	SJ6	234	

Supplementary Table 1

The table describes the body weight of the rats (n=6) during the habituation procedure and the auditory fMRI acquisition phase. No signs of abnormal weight gain or loss was evident.
