# Supplementary material for: Gradual Restraint Habituation for Awake Functional Magnetic Resonance Imaging Combined With a Sparse Imaging Paradigm Reduces Motion Artifacts and Stress Levels in Rodents
Source: Front Neurosci. 2021 Dec 21;15:805679. doi: 10.3389/fnins.2021.805679 (PMC8724036; doi:10.3389/fnins.2021.805679)
Supplement: Supplementary file 2 [file Table_2.pdf]

Animal	Habituation phase	FCM (ng/g)	S.E.M.
Rat1	Pre-handling	84.455	0.2
Rat2	Pre-handling	88.80367	1.2
Rat3	Pre-handling	96.00333	0.8
Rat4	Pre-handling	95.60933	0.2
Rat5	Pre-handling	95.467	1.4
Rat6	Pre-handling	94.195	1.4
Rat1	Handling acute	70.84333	0.3
Rat2	Handling acute	67.918	1.2
Rat3	Handling acute	61.697	0.1
Rat4	Handling acute	67.99967	0.8
Rat5	Handling acute	76.713	0.3
Rat6	Handling acute	72.629	1.2
Rat1	Handling chronic	73.56	0.3
Rat2	Handling chronic	61.732	0.4
Rat3	Handling chronic	61.843	1.3
Rat4	Handling chronic	60.50467	0.1
Rat5	Handling chronic	62.12367	0.8
Rat6	Handling chronic	70.68967	0.4
Rat1	Body restraint acute	89.27	0.6
Rat2	Body restraint acute	92.17833	0.8
Rat3	Body restraint acute	99.70133	1.5
Rat4	Body restraint acute	101.877	0.7
Rat5	Body restraint acute	92.14667	0.3
Rat6	Body restraint acute	96.79167	1.2
Rat1	Body restraint chronic	35.74533	0.3
Rat2	Body restraint chronic	38.42167	0.4
Rat3	Body restraint chronic	43.032	0.7
Rat4	Body restraint chronic	41.17267	0.8
Rat5	Body restraint chronic	44.58133	1.2
Rat6	Body restraint chronic	38.714	0.4
Rat1	Darkness acute	116.5777	2.4
Rat2	Darkness acute	101.752	0.6
Rat3	Darkness acute	118.453	2.5
Rat4	Darkness acute	105.649	0.6
Rat5	Darkness acute	121.8613	2.6
Rat6	Darkness acute	107.2447	0.8
Rat1	Darkness chronic	44.27633	0.5
Rat2	Darkness chronic	48.58433	0.8
Rat3	Darkness chronic	50.49767	1.0
Rat4	Darkness chronic	48.49133	0.8
Rat5	Darkness chronic	47.87567	0.6
Rat6	Darkness chronic	43.43633	0.9

Rat1	MRI noise acute	44.2400	2.2
Rat2	MRI noise acute	43.7400	0.5
Rat3	MRI noise acute	42.9490	1.3
Rat4	MRI noise acute	40.1307	0.7
Rat5	MRI noise acute	41.7900	0.7
Rat6	MRI noise chronic	40.0300	0.5
Rat1	MRI noise chronic	31.5023	0.1
Rat2	MRI noise chronic	24.7913	0.2
Rat3	MRI noise chronic	31.5060	0.1
Rat4	MRI noise chronic	27.0750	0.4
Rat5	MRI noise chronic	27.4450	0.8
Rat6	MRI noise chronic	30.1740	0.1
Rat1	Head fixation acute	220.8250	2.4
Rat2	Head fixation acute	225.9720	4.0
Rat3	Head fixation acute	213.8687	1.5
Rat4	Head fixation acute	221.9110	1.7
Rat5	Head fixation acute	205.3030	3.0
Rat6	Head fixation acute	207.2540	1.4
Rat1	Head fixation chronic	71.1210	0.5
Rat2	Head fixation chronic	68.9370	0.9
Rat3	Head fixation chronic	69.1270	0.4
Rat4	Head fixation chronic	65.9930	0.4
Rat5	Head fixation chronic	69.9840	0.8
Rat6	Head fixation chronic	75.5097	0.6
Rat1	MRI acquisition acute	56.1333	1.6
Rat2	MRI acquisition acute	40.7733	1.2
Rat3	MRI acquisition acute	37.1000	1.3
Rat4	MRI acquisition acute	69.2333	3.8
Rat5	MRI acquisition acute	36.6067	0.8
Rat6	MRI acquisition acute	78.0067	0.9
Rat1	MRI acquisition chronic	37.6127	3.5
Rat2	MRI acquisition chronic	33.4767	2.9
Rat3	MRI acquisition chronic	49.8667	1.5
Rat4	MRI acquisition chronic	63.1800	3.2
Rat5	MRI acquisition chronic	26.5573	1.4
Rat6	MRI acquisition chronic	39.1727	3.0

Supplementary Table 2

Corticosterone levels were monitored by analyzing fecal corticosterone metabolite (FCM) concentrations of the stress hormone. The table reports the FCM levels in ng /g ± standard error of the mean (S.E.M) for each individual rat during all stages of habituation. Values are rounded up to two decimal places. The conditions reported in the table correspond to the timeline reported in Figure 1A.
